# Associations between preoperative weight and body composition changes and surgical outcomes in patients with obesity and gastrointestinal cancer: a protocol for a prospective observational study in the active together cancer prehabilitation service

**DOI:** 10.1136/bmjopen-2026-120499

**Published:** 2026-06-18

**Authors:** Scott Newbould, Anna Myers, Kerry Rosenthal, Keith Harris, Michael Thelwell, Robert Copeland, Sade Allott

**Affiliations:** 1 Advanced Wellbeing Research Centre, Sheffield Hallam University, Sheffield, England, UK; 2 Physical Activity, Wellbeing and Public Health Research Group, School of Sport and Physical Activity, Sheffield Hallam University, Sheffield, UK; 3 School of Computing and Digital Technologies, Sheffield Hallam University, Sheffield, UK; 4 Sheffield Teaching Hospitals NHS Foundation Trust, Sheffield, UK

**Keywords:** Body Mass Index, Gastrointestinal tumours, Colorectal surgery, Adult oncology, Physical Fitness, Quality of Life

## Abstract

**Abstract:**

**Introduction:**

The impact of preoperative weight loss and body composition changes on surgical and patient-reported outcomes remains unclear in gastrointestinal cancer patients. Prehabilitation programmes integrating exercise, nutrition and psychological support can improve surgical readiness and recovery but the role of body mass and composition changes within such services is not well understood. This study aims to investigate the associations between preoperative changes in body mass and composition and surgical, physical fitness, nutritional and quality of life outcomes among people with obesity undergoing surgery for upper gastrointestinal or colorectal cancer within the context of a cancer prehabilitation service (Active Together).

**Methods and analysis:**

This prospective observational study will recruit 100 adults (≥18 years; body mass index ≥30 kg/m²) scheduled for curative upper gastrointestinal or colorectal cancer surgery and enrolled in the Active Together prehabilitation service. Participants will attend two study visits: one as soon as possible after diagnosis and one within 2 weeks before surgery. Participants will undergo body mass, composition and size measurements and complete questionnaires on their nutritional status and quality of life. Routinely collected surgical outcomes (complications, operative approach and duration, length of hospital stay, readmissions, 1-year survival) and Active Together assessment data (physical fitness, psychological well-being, nutritional status) will also be collected. Correlation analyses and regression models will be used to explore the associations between preoperative changes in body mass and composition and surgical, physical fitness, nutritional status and quality of life outcomes.

**Ethics and dissemination:**

Ethical approval has been obtained from the Health Research Authority (Integrated Research Application System project ID 361634; Research Ethics Comittee reference 26/YH/0019). Written informed consent will be obtained from all participants. Data will be processed in accordance with General Data Protection Regulations and the Data Protection Act 2018. Findings will be disseminated via peer-reviewed publications, conference presentations and patient and public involvement activities.

**STRENGTHS AND LIMITATIONS OF THIS STUDY**

- The observational nature of the study provides an ecologically valid insight into patient outcomes in an evidence-based cancer prehabilitation service.
- Longitudinal assessment of body mass and composition, surgical outcomes, physical fitness, nutritional status and quality of life allow investigation of the relationships between the changes in each metric across the perioperative period.
- The observational nature of the study precludes the ability to draw causal inferences.
- The potential heterogeneity of participant characteristics and treatment pathway may confound the associations reported; however, these will be accounted for statistically where possible.

## Introduction

 Gastrointestinal (GI) cancers are a leading cause of global morbidity and mortality^1 2^ and induce significant costs to health services.^3^ Surgical intervention (eg, resection) is a primary treatment for GI cancers.^4^ However, surgery carries the risk of complications, risks which are increased for individuals with obesity.^5 6^ Consequently, patients with obesity are advised to lose weight prior to surgery.^7^ Previous research has shown that weight loss is feasible in a pre-surgery context for cancer patients^8^ and does not lead to increases in length of stay or postoperative complications.^9 10^ Indeed, a recent randomised controlled trial feasibility study investigating intentional pre-surgery weight loss in colorectal cancer patients reported no difference in complications between the weight loss and usual care group, and that those who lost more than 3.2% of their body weight had a 50% reduction in surgical complications.^11^

However, although weight loss may make the surgical procedure easier for surgeons to perform and/or reduce the risk of surgical complications, the effects of weight loss must be viewed within the context of the whole cancer patient journey. Indeed, greater preoperative weight loss is associated with worse long-term outcomes such as mortality and disease recurrence in upper GI and colorectal patients.^1012^,^14^ A possible explanation is that weight loss prior to surgery, particularly rapid weight loss, may result in the reduction of not only fat mass but also muscle mass, which is associated with increased mortality^15^ and is linked to metabolic disease.^16^ Additionally, low muscle mass has been associated with post-operative complications in cancer patients undergoing gastric surgery.^17^ Estimates suggest that one in two people with cancer will experience cachexia,^18 19^ which causes approximately 20–30% of all deaths in cancer patients.^20 21^ Consequently, interventions with the potential to exacerbate cachexia require thorough evaluation prior to adoption.

Therefore, presurgery weight loss offers potential benefits and drawbacks that may be dependent on the actual body composition changes that occur; lower fat mass might be beneficial for reducing surgical complications, but reduced muscle mass is likely detrimental for overall morbidity and mortality. Until the effects of preoperative weight loss are better understood, it may not be appropriate to encourage patients to lose weight.^22^ A greater understanding of these factors would help to generate evidence-based recommendations for cancer patients providing much needed guidance for dietitians and specialist exercise professionals working with people affected by cancer.

Current recommendations advocate for prehabilitation programmes for cancer patients,^23^ which is the use of targeted interventions between the time of diagnosis and acute treatment to promote health and reduce the risk of future impairments.^24^ Prehabilitation interventions including exercise, nutrition and psychological support (either alone or in combination) have been shown to improve patient outcomes such as surgical complications, length of hospital stay and quality of life compared with usual care, and thus should be considered in standard care.^25^ Indeed, the National Cancer Plan for England acknowledges the importance of cancer prehabilitation and sets out the ambition to deliver a national ‘digital first’ prehabilitation offer by 2028.^26^ Therefore, investigating the effects of preoperative weight loss within a prehabilitation service will provide valuable information that could benefit individuals within this type of service.

Active Together is a multimodal prehabilitation and rehabilitation service in South Yorkshire, UK, which provides support to cancer patients to prepare for and recover from treatment.^27^ The service, which was co-designed by cancer patients, clinicians, professionals and academics, provides exercise, nutrition and psychological support to patients throughout the cancer care journey, spanning from prehabilitation before and during treatment to restorative and supportive rehabilitation after treatment.^28^ Patients are referred to the service after diagnosis where they undergo assessments to ascertain their physical, nutritional and psychological status, before receiving person-centred support underpinned by behaviour change techniques.^28^

The measures of physical function, dietetic need and psychological well-being that are routinely collected by Active Together are useful for understanding patient experience and outcomes throughout their cancer journey. However, the impact of preoperative changes in body weight and composition on these measures is currently unknown. Therefore, combining the primary data from this study with the data collected by the Active Together service will provide a rich and novel insight into patient outcomes in response to surgery within the context of body mass and composition changes.

The aim of this study is therefore to investigate the associations between preoperative body mass and composition changes and surgical outcomes, physical fitness, nutritional status and quality of life in patients living with GI cancer and obesity within a (p)rehabilitation context.

## Methods and analysis

### Study design

This is a prospective observational study to assess changes in patient body weight and composition during the preoperative phase of GI cancer treatment and the associations these have with surgical outcomes, physical fitness, nutritional status and quality of life. Recruitment and data collection for this study will take place between April 2026 and February 2027. Participants will complete two data collection sessions for this study: once as soon as possible after diagnosis and once within the 2 weeks prior to surgery. The time between the first and second visits will vary depending on tumour location, wait times for surgery and length of neo-adjuvant treatments (if applicable).

**Figure 1:**
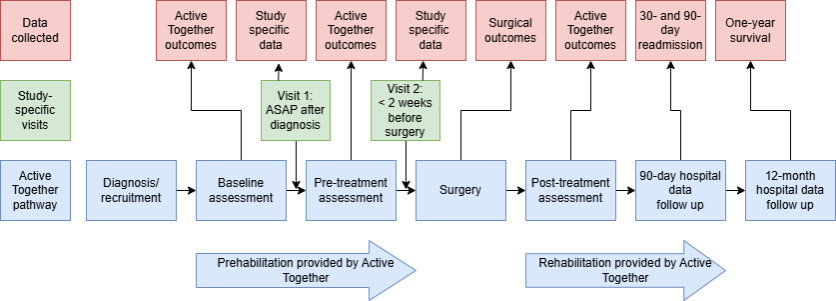
Schematic of data collection in relation to study-specific visits, surgery, and the Active Together assessment pathway. Data will be collected by the Active Together service at baseline, pretreatment and post-treatment, and at 90 days post treatment. Active Together define treatment to include additional forms of acute treatment, for example chemo or radiotherapy, and so pretreatment and post-treatment assessments may occur at varying intervals from surgery. Study-specific data will be collected twice: once as soon as possible after diagnosis, and once within the 2 weeks prior to surgery. Due to differences in treatment pathways and surgery waiting times, the duration between visit 1 and visit 2 will vary between participants, as well as the duration between Active Together assessments. Surgical data will be collected from the surgical team after patient discharge. The study specific, surgical and Active Together outcomes are outlined in the Procedures section.

As well as these two data collection sessions, participants will continue to receive their usual care from the Active Together (p)rehabilitation service, which includes assessments at pre-defined time points throughout their cancer care journey. The data collected from these assessments will be used for this study, as well as surgical data that will be collected from the surgical team and hospital records. Figure 1 provides an overview of the timing and type of data collected from each source, and the Procedures section details the outcome measures collected from each source.

### Participants

This study will recruit participants from the South Yorkshire Active Together (p)rehabilitation service who are 18 years or older, have a body mass index (BMI) of ≥30 kg/m^2^ and are candidates for curative upper GI or colorectal surgery as part of their cancer treatment. Exclusion criteria are pregnancy and those who are unable to complete the study procedures or provide informed consent (lack capacity). Prospective participants will be screened for eligibility by checking the patient’s age, cancer type, treatment plan and BMI. All individuals who meet the eligibility criteria will be provided information about the study by clinicians in routine Active Together sessions and their details passed to the research team with the prospective participant’s consent. Alternatively, potential participants will be contacted directly by a member of their clinical team. Participants will be reimbursed for their travel and will be given a £25 voucher on completion of the second study visit.

### Procedures

#### Primary data

Participants will be required to attend the Advanced Wellbeing Research Centre (AWRC) at Sheffield Hallam University on two separate occasions. The first visit will take place as soon as possible after diagnosis, during which participants will complete two questionnaires and four physical assessments. On arriving at the AWRC, participants will be greeted by a member of the research team and taken to the morphology lab where they will be seated in a neutral space with no sight of the equipment. The researcher will explain the study again and provide an opportunity to ask any questions before the participant provides their informed consent to participate.

Participants will then be asked to complete the following questionnaires: the EuroQol 5-Dimensions 5-Levels (EQ-5D-5L) to measure quality of life^29^ and the Patient Generated Subjective Global Assessment Short Form (PG-SGA-SF) to measure nutritional status.^30 31^ Next, participants will be asked to void their bladder, change into form-fitting, non-compressive clothing, and remove all jewellery to complete the four physical assessments: stature (Seca 264, Seca, Germany), body mass and body composition (proportions of fat and fat-free mass) using a BOD POD (Cosmed, Italy) and body size and shape (including waist, upper arm, and calf circumference) using the Size Stream SS20 3D body scanner (Size Stream, NC, USA). The second visit will take place within the 2 weeks prior to the participant having surgery, and the data collection for this visit will be the same as visit one, with the addition of another questionnaire to gather information on participants’ weight loss intentions, strategies and advice received; see online supplemental material 1.

For the body composition and size measurements, participants will wear form-fitting, non-compressive clothing and a swim cap. Additionally, participants will be asked to refrain from eating or exercising within the 2 hours prior to the visit and to arrive in a normal state of hydration (ie, not drinking any more or less than they usually do). If a medical condition precludes a participant from fasting, they will be instructed to record what they eat before the session and to replicate this for the second session. The BOD POD estimates body composition using air displacement plethysmography, for which the procedure requires participants to be weighed on a set of calibrated scales and then sit inside the pod for approximately 3 min (two or three 45-second measurements). If participants are unwilling or unable to use the BOD POD, they will instead have their body composition measured using a set of bioelectrical impedance scales (TANITA MC-780MA-N S, TANITA Europe, Netherlands). The Size Stream SS20 procedures require participants to stand still inside the device for approximately 20 s (a single 6-second measurement).

For participants that are eligible and wish to volunteer to take part in the study but express that they are unable to attend the AWRC site, a member of the research team will conduct the two data collection sessions where the participant attends Active Together sessions. These sessions will not include BOD POD and Size Stream measurements and instead only include body mass and composition measurement performed using the bioelectrical impedance scales and questionnaires. These participants will be included in the study to maximise recruitment and the amount of data collected, as well as attenuating the bias of the data towards participants that are more mobile/have access to transport/live closer to the AWRC.

#### Secondary data

In addition to the outcomes detailed above that will be gathered by the research team, data will be collected as part of the participants’ usual care from two other sources: from their NHS records for surgical information, hospital length of stay, readmissions and 1-year survival, and from the Active Together service for physical fitness and questionnaire assessments (table 1). These outcomes will be measured at the time points outlined in figure 1. The physical fitness outcomes from Active Together will be collected by members of the Active Together team, with the procedures used to collect this data provided in online supplemental material 2.

**Table 1:** Data collected by the hospital and Active Together.

Hospital data	Active Together outcomes	
Surgical outcomes	Physical fitness	Questionnaires
Clavien-Dindo classification of postoperative surgical complications	6-minute walk test distance (m)	Patient-Generated Subjective Global Assessment Short Form
Type of surgical complications	60-second sit-to-stand repetitions	Quality of life: EuroQol 5-Dimensions 5-Levels
Length of operation time	2-minute step test repetitions	Fatigue: Functional Assessment of Chronic Illness Therapy-Fatigue
Surgical approach	Handgrip strength (kg)	Anxiety: General Anxiety Disorder 7
Length of critical care stay post-surgery	Blood pressure (mm Hg)	Depression: Patient Health Questionnaire 9
Length of hospital stay post-surgery	Physical activity level (exercise vital signs)	Self-Efficacy for Exercise Scale
Readmissions within 30 and 90 days (and reason for this)		
1-year survival		
Treatment pathway		

### Patient and public involvement

The study proposal was presented to the Public Involvement in Research Group at Sheffield Hallam University who provided feedback on the study plan and recommendations about how to improve the participant experience during the study. In addition, two patient and public focus groups will be held for the project which will include patients, service users, and/or their carers and members of the public. The first has taken place prior to the start of the study and sought input on the study design, procedures and participant-facing documentation. The second will take place after data collection and analysis is complete and will seek input on dissemination materials.

This project also has a steering committee which includes two members of the public (one is a former Active Together patient who has experience of the pathway) as well as academics and clinicians with expertise in cancer and prehabilitation. The steering group will convene twice a year to review study progress and ensure the study is conducted—and the data are generated, documented and reported—in accordance with the protocol, good clinical practice and the applicable regulatory requirements.

### Statistics and analysis

**Figure 2:**
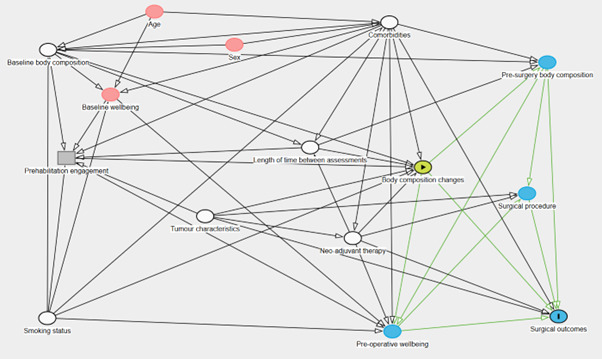
Directed acyclic graph for investigating the effect of body composition changes on surgical outcomes. Baseline and preoperative well-being include nutritional status, psychological well-being and physical fitness.

Directed acyclic graphs have been created for the outcome measures in this study to identify confounding factors and determine which variables to adjust for during analysis. Figure 2 shows the causal pathway and potential confounders for investigating the effect of body composition changes on surgical outcomes. Online supplemental material 3 includes directed acyclic graphs for preoperative well-being, post-treatment well-being and 1-year survival. All quantitative data will be treated as continuous data, excluding Clavien-Dindo classification of postoperative surgical complications (ordinal) and the surgical approach, type of surgical complications and treatment pathway (nominal).

Pearson (or Spearman, depending on the distribution of the data) correlation coefficients will be calculated to explore associations between outcome measures (eg, body weight, size and shape, body composition, body mass change, PG-SGA-SF, EQ-5D-5L, etc) and clinical outcomes (eg, surgical complications, length of stay, number of emergency readmissions and length of operating time). P values <0.05 will be considered statistically significant. Linear and multivariate regression models will then be used to investigate the strength of associations between preoperative changes in patient body measures (eg, body weight and composition), self-reported outcomes (ie, questionnaire data) and clinical outcomes. Additionally, exploratory analyses comparing outcomes between groups of participants (eg, those that lose weight vs those that do not) will be completed, with groups created using thresholds used in the previous literature^10^ and/or data-driven approaches such as a median split^11^ or optimal cut-point determination^32^.

Separate linear regression models will be created to investigate the amount of variation in self-reported and clinical outcomes which are explained by preoperative changes in patient body measures. Then, multivariate regression models will be created which combine patient outcome measures that contribute significantly to the estimation of clinical outcomes. Ordinal logistic regression models will also be used to explore the relationship between patient outcome measures and ordinal clinical outcome variables, such as surgical complications (Clavien-Dindo classification) and the surgical approach used. Where the distribution of data indicates it would be appropriate (ie, for non-linear relationships between predictor and outcome variables), other regression approaches such as restricted cubic splines or generalised additive models will be used. Regression analyses are appropriate as this is exploratory research, and this approach allows the observation of relationships between all variables. The regression models will all be adjusted for the variability in time between the assessment points, tumour group and pretreatment therapies where these are shown to reduce residual variance and not introduce collinearity to the model. Any outliers will be investigated to ensure that they are legitimate observations; only implausible data will be removed.

Multiple regression models will be assessed for multicollinearity between input variables using variance inflation factor and tolerance collinearity statistics and for independence of errors using the Durbin-Watson test statistic. If the largest variance inflation factor value is >10, or tolerance values are below 0.2 this could indicate potential issues in the model associated with multicollinearity. Also, if Durbin-Watson values differed significantly from two this would suggest dependence of errors between input variables in the model. Additionally, regression assumptions (eg, homoscedasticity, normally distributed errors) will be checked via diagnostic plots, and transformations of variables will be used if assumptions are not met.

Data from the Active Together service shows that many patients are unable to attend assessment appointments prior to surgery, often due to short notice of surgery dates. This might lead to missing data at the presurgery assessment point (visit 2). All efforts will be made to make attending appointments as convenient as possible for patients. Additionally, patients are routinely weighed before surgery; therefore, the key outcomes of body mass change and surgical outcomes should always be available. Therefore, statistical analyses for body mass change will include all participants, and analyses including body composition, size and shape, and questionnaire data will be performed on a smaller cohort if some data are missing.

Complete case analyses will be performed when covariates (eg, body composition variables) contain missing data. Complete case analysis can lead to biased parameter estimates in regression models if the data are not missing completely at random (MCAR). Therefore, graphical techniques and Little’s hypothesis test of the MCAR assumption will be used to explore whether the MCAR assumption is a reasonable one to make with the collected data. If not, a standard multiple imputation method will be implemented (eg, Multiple Imputation by Chained Equations, Linear Regression Imputation or Predictive Mean Matching).

For linear regression analyses, assuming a medium effect size (F^2^=0.15), significance level=0.05, power=0.8 and number of predictors=6, a sample size of 98 is required.^33^ For correlation, assuming a two-tailed test, a medium effect size (rho=0.3), significance level=0.05 and power=0.8, a sample size of 84 is required.^33^ Therefore, a sample size of 100 participants is required to detect medium effect sizes with acceptable statistical power. Furthermore, 100 participants is a reasonable estimate of the number of potential participants within the study’s time frame based on the demographics of current and previous Active Together patients (ie, number of patients awaiting surgery for upper GI or colorectal cancer with a BMI ≥30 kg/m^2^).

For the data collected through the weight loss intention, strategies and advice questionnaire, the number and proportion of responses for each question will be recorded and subgroup analyses performed depending on the responses, for example comparisons of surgical outcomes between those who lost weight intentionally, those who lost weight unintentionally and those who didn’t lose weight. The final question will be analysed using thematic analysis by manually identifying patterns and themes in the responses and will be reported narratively. Member checking will be undertaken by an independent qualitative researcher to check that the analysis is accurate and has not over-represented or under-represented any aspects of the data.

### Ethics and dissemination

The Principal Investigator will ensure that this study is conducted in accordance with the principles of the Declaration of Helsinki, relevant regulations and with Good Clinical Practice. This study has been approved by the Health Research Authority (Integrated Research Application System project ID 361634; Research Ethics Committee reference 26/YH/0019). The Principal Investigator will submit and, where necessary, obtain approval from the Health Research Authority for any amendments to the original approved documents.

Prospective participants will be provided with a copy of the Participant Information Sheet which details the nature of the study, what will be required of them, the benefits and risks of their participation and information on how their data will be used. Written informed consent will be provided by all participants at the start of their first visit for the study, before any study-related procedures take place. This will be obtained in the form of a participant-signed and dated informed consent form, including the dated signature of the person obtaining informed consent. The Participant Information Sheet and all other participant-facing documentation has been scrutinised and deemed appropriate by the Public and Patient Involvement steering committee.

A participant may choose to withdraw early from the study at any time. If a participant withdraws from the study before their second visit or surgery, their data will also be removed from the study dataset as both visits and surgical data are required to answer the research questions. If a participant withdraws from the study after their surgery, no further data will be collected from their records (surgical or Active Together outcomes) for the study, but data that has already been collected (ie, from surgery and the two data collection sessions) will be used for analysis. Participants will be made aware of their right to withdraw and what happens to their data on withdrawal via the Participant Information Sheet and give consent to this on the Informed Consent Form.

Data will be input into spreadsheets by the research team and held on a secure research drive by Sheffield Hallam University. Sheffield Hallam University policy requires study data to be archived for 5 years; this will be made clear on the Participant Information Sheet, and participants will consent to storing data in this way in the Informed Consent Form. Participants will also be asked for their consent for their anonymised data to be stored in the Sheffield Hallam University Research Data Archive (SHURDA) database for a period of 10 years since the last time any third party has requested access to the data. If participants do not consent to this, their data will not be uploaded to SHURDA and will be destroyed after 5 years.

The study will comply with the General Data Protection Regulation and Data Protection Act 2018, which require data to be deidentified as soon as it is practical to do so. The processing of the personal data of participants will be minimised by using a unique participant study ID on all study documents and any electronic database(s). A password-protected ‘key’ file containing participant information and their study ID will be kept separate to all study documentation. All documents will be stored securely and only accessible by study staff and authorised personnel. The study staff will safeguard the privacy of participants’ personal data.

## Supplementary material

10.1136/bmjopen-2026-120499 online supplemental file 1

## Data Availability

No data are available.
