# Supplementary material for: Associations between preoperative weight and body composition changes and surgical outcomes in patients with obesity and gastrointestinal cancer: a protocol for a prospective observational study in the active together cancer prehabilitation service
Source: BMJ Open. 2026 Jun 18;16(6):e120499. doi: 10.1136/bmjopen-2026-120499 (PMC13289135; doi:10.1136/bmjopen-2026-120499)
Supplement: online supplemental file 1 [file bmjopen-16-6-s001.docx]

**Supplementary material 1 – Data Collection Questionnaires**

EQ-5D-5L


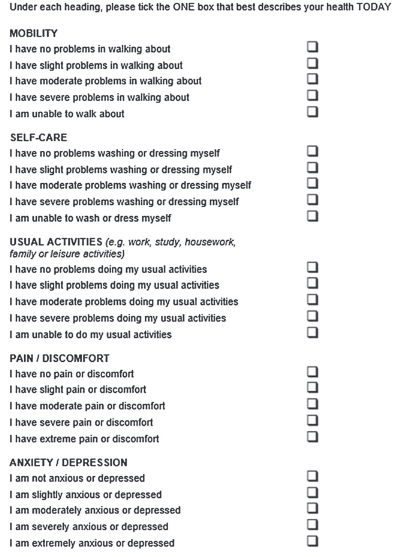


PG-SGA-SF


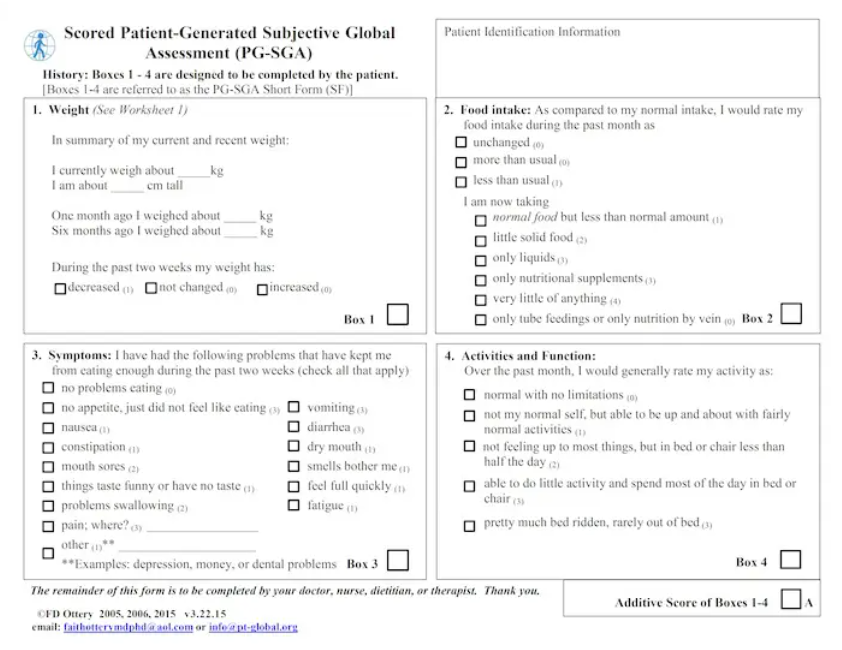


Weight loss intentions, strategies, and advice questionnaire

1. Have you been advised to lose weight for your surgery?

- Yes
- No
- Don’t know

1. If yes to question 1, who advised you to lose weight? Tick all that apply.
   - Surgeon
   - Oncologist
   - Dietitian
   - Clinical nurse specialist
   - GP
   - Any other health care professional
   - Family member
   - Internet/online/news
   - Other:

______________________________________________________________________________________________________________________________________________

1. Did you try to lose weight on purpose before surgery?

- Yes
- No
- Don’t know

1. If yes to question 3, what methods did you use to try and aid weight loss? Tick all that apply.

- General healthy eating (covers low fat/low sugar/portion control/balanced diet)
- Low carbohydrate/high protein
- Fasting
- Calorie restriction
- Increased physical activity
- Weight loss medication or jabs
- Weight management groups (such as Weight Watchers, Slimming World)
- Other:

______________________________________________________________________________________________________________________________________________

1. Have you been advised to follow any specific diets in preparation for your surgery or due to the location of the tumour?
   - Yes
   - No
   - Don’t know
2. If yes to question 5, who gave you advice? Tick all that apply.
   - Surgeon
   - Oncologist
   - Dietitian
   - Clinical nurse specialist
   - GP
   - Any other health care professional
   - Family member
   - Internet/online/news
   - Other:

______________________________________________________________________________________________________________________________________________

1. If yes to question 5, what did they advise? Tick all that apply.
   - General healthy eating (covers low fat/low sugar/portion control/balanced diet)
   - Low carbohydrate/high protein
   - Fasting
   - Calorie restriction
   - Increased physical activity
   - Weight loss medication or jabs
   - Weight management groups (such as Weight watchers, Slimming World)
   - Low fibre/low residue diet
   - Soft/liquid diet
   - Stent-specific advice
   - Other:

______________________________________________________________________________________________________________________________________________

1. If yes to question 5, were you able to follow this advice?
   - Yes
   - No
   - Partially
2. If yes to question 8, what made it easier or harder to follow the advice you were given?

____________________________________________________________________________________________________________________________________________________________________________________________________________________________________________________________________________________________________________

1. When thinking about the advice and care you received from healthcare professionals regarding your body weight in the time since your diagnosis, what feedback would you give (to healthcare professionals) about your experience?

____________________________________________________________________________________________________________________________________________________________________________________________________________________________________________________________________________________________________________

**Supplementary material 2 - Description of Active Together Data Collection Procedures**

The Active Together assessments will be carried out by the Active Together team, either by a registered physiotherapist, clinical exercise physiologist, or a Level 3/4 Personal Trainer. All individuals conducting these assessments have received the same extensive training and follow the same protocols, outlined below.

Six-minute walk test

The six-minute walk test is a valid test of functional exercise capacity in both cancer patients (Schmidt et al., 2013) and those with obesity (Larsson and Reynisdottir, 2008).

Protocol:

1. The test should be completed over a measured track. Consistency of measurement is very important to ensure accuracy of test results.
2. Prior to walking say to patient: The objective of this test is to walk as FAR AS POSSIBLE for 6 minutes. You will walk back and forth along this course (walking around the cones and keeping as close to the cones as possible; demonstrate one lap) for six minutes. You may slow down if necessary. If you stop, I want you to continue to walk again as soon as possible. Please only walk, do not run even if you feel you can. You will be informed of the time and encouraged each minute. Please do not talk during the test unless you have a problem, or I ask you a question. You must let me know if you have any chest pain or dizziness. When six minutes is up, I will ask you to STOP where you are. Do you have any questions?
3. To begin say to patient: Start now, or whenever you are ready (start stopwatch when walking starts).
4. During the test: Provide the following standard encouragements in even tones. Do not use other words of encouragement or body language to speed up.

- At 1 minute: You are doing well. You have 5 minutes to go.
- At 2nd minute: Keep up the good work. You have 4 minutes to go.
- At 3rd minute: You are doing well. You are halfway done.
- At 4th minute: Keep up the good work. You have only 2 minutes left.
- At 5th minute: You are doing well. You have only 1 minute to go.
- At 6th minute: Please stop where you are.

1. If the patient stops during the test: Allow the patient to rest or sit in a chair if they wish, and check oxygen saturation and heart rate. Ask the patient why they stopped. Keep the stopwatch running and advise: Please resume walking whenever you feel able.
2. At the end of the test record the total distance walked.

Two-minute step test protocol

1. The two-minute step test is a sub-maximal measure of cardiorespiratory fitness and can be used as an alternative to the six-minute walk test.
2. The equipment needed for this test is a tape measure, a set of hurdles, a tally counter, a countdown timer with an audible alarm, a wipeable marker pen, and a heart rate monitor.
3. To begin, set up two vertical poles by inserting them into the rubber bases.
4. The test is individualised based on height using two reference points: the pelvis and the knee.
5. For the pelvis measurement, either the anterior superior iliac spine (ASIS), which is the bony process at the front of the hip, or the top of the iliac crest (Figure 1). Ideally choose the ASIS, but if this is difficult to palpate, choose the iliac crest instead.


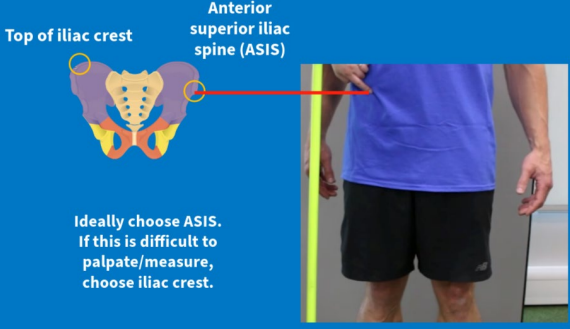


*Figure 1. Anterior superior iliac spine (ASIS).*

1. The second measurement should be at the top edge of the kneecap (Figure 2).


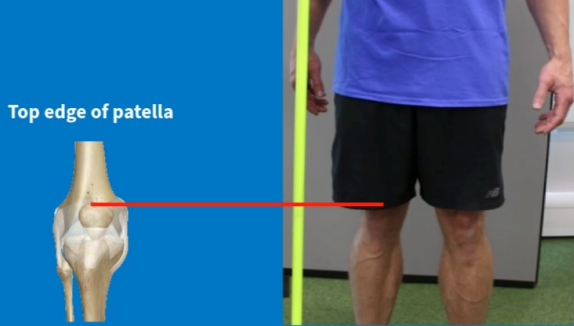


*Figure 2. Top of patella.*

1. Using a wipeable marker, mark the pole in line with the pelvis and the knee.
2. Measure and mark the midpoint between the pelvis and the patella.
3. Measure the distance from the base to the midpoint (Figure 3).


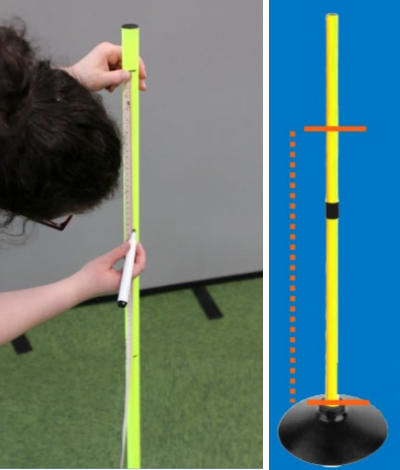


*Figure 3. Marking and measuring the poles for the two-minute step test.*

1. Record the midpoint height and use this for all further tests for that participant.
2. Mark a line on the second pole at the same height. It's important that the height is measured from exactly the same point from the rubber base.
3. Attach one black clip to each vertical pole, ensuring that the top edge of the clip is in line with the midpoint line, then attach the horizontal pole to each clip.
4. Ask the patient to stand with their right side facing you.
5. If concerned about a patient’s balance, position the equipment close to a handrail or a wall, or ask an extra staff member to spot them. The participant is allowed to hold/lean on something for balance during the test if needed.
6. Ask the participant to raise their knees above the pole, aiming for as many steps as possible in 2 minutes.
7. If the top of the knee is in line with the horizontal pole this counts as a step (Figure 4).
8. Before starting the test, set a 2-minute timer alarm.
9. Use the tally counter to count each time the right knee crosses the line. Do not count the left knee.
10. If a patient is consistently not crossing the line, it is OK to give feedback but try to keep encouragement to a minimum.


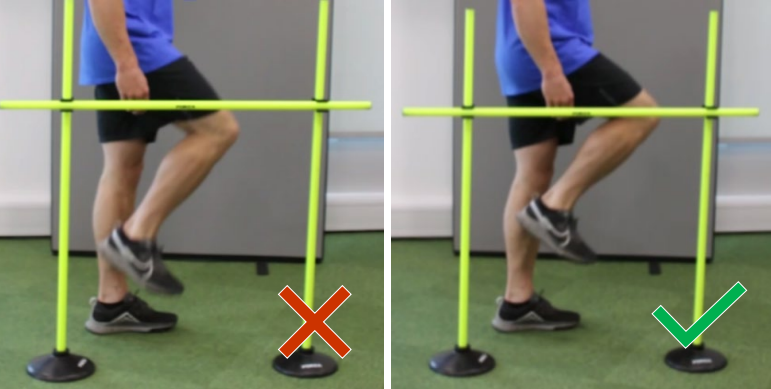


*Figure 4. Knee height for the two-minute step test.*

1. If a patient feels unwell during the test, ask them to stop. Monitor their recovery and take appropriate action depending on symptoms.
2. Record the total number of times their right knee reached the horizontal pole in two minutes.
3. This is a sub-maximal exercise test. If a patient’s heart rate exceeds 85% HRmax, they should be asked to slow their pace. If after 15 seconds, heart rate does not decline below 85% HRmax, ask the patient to stop the test. If a heart rate monitor is not available, the RPE scale and Talk Test can be used as an alternative. Whilst it is normal for patients to feel out of breath during the test, patients should still be able to talk (rapid, heavy breathing should be avoided). In addition, patients should not exceed 17/20 RPE (very hard). Two-minute step test script: “The aim of this test is to perform as many steps as possible in two minutes. Each time your knee crosses the line, this will be counted as a step. Please step at a consistent pace that you think you can maintain for two minutes. It is normal to feel out of breath during this test. However, if your breathing rate increases to the point where you feel you could not speak, please slow down. Please do not run on the spot – one foot must remain on the floor. If you feel tired, you may stop if necessary, but I want you to start again as soon as you feel ready. If you feel unwell at any point during the test, please stop. Do you have any questions?”

Sit-to-stand protocol

1. The sit-to-stand test is administered using a chair without arms, with seat height of 42-46cm. Ensure that the same chair height is used for all tests.
2. The chair, with rubber tips on the legs, should be placed against a wall to prevent it from moving.
3. The participant is seated in the middle of the chair, back straight; feet approximately a shoulder width apart and placed on the floor at an angle slightly back from the knees, with one foot slightly in front of the other to help maintain balance.
4. Arms should not be used to assist the sit-to-stand. Arms should be crossed and held against the chest during the test.
5. Demonstrate the task both slowly and quickly.
6. Have the patient practice a repetition or two before completing the test.
7. If a patient must use their arms to complete the test, they are scored 0.
8. The patient is encouraged to complete as many full stands as possible within 60 seconds.
9. The patient is instructed to fully sit between each stand.
10. While monitoring the participant’s performance to ensure proper form, the tester silently counts the completion of each correct stand, noting the number of completed repetitions at both 30 seconds and 60 seconds.
11. More than halfway up at the end of 30 seconds and 60 seconds counts as a full stand. Incorrectly executed stands are not counted.
12. This should be completed with no encouragement by the tester.

Grip strength protocol

- 1. Record the participant’s hand dominance.
  2. Demonstrate how to hold the dynamometer to the participant by testing it on yourself and explain how the dial registers the best result by squeezing as tightly as possible.
  3. Sit them comfortably in a chair with a back support.
  4. Ask the participant to position the arm at a right angle and the elbow by the side of the body and keep their feet flat on the floor. Ask the participant to roll their trousers/jeans up to ensure their feet are flat on the floor and do not rise from the floor when squeezing the dynamometer – see Figure 5.


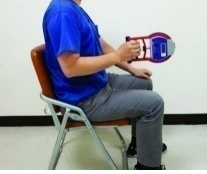


*Figure 5. Hand grip dynamometer body positioning*

- 1. Their wrists should be just over the end of the chair’s arm, thumb facing upwards.
  2. Ask them to position their thumb round one side and their fingers around the other side of the handle. When they are holding the dynamometer in the correct position their fingers and thumb should be visible on the same side of the apparatus.
  3. Check with them that the instrument feels comfortable in their hand. The position of the handle can be adjusted if necessary for different sized hands. Whether the handle needs altering is based on the distance of the four fingers from the palm of the hand. If the fingernails are digging into the palm, it will be uncomfortable for the participant and means that the handle needs moving further away from the mechanism. If it looks as though the fingers are not close enough to the palm and it feels to the participant as though their hand may slip off the handle when squeezing, it suggests that the handle needs to be adjusted to bring it closer to the mechanism.
  4. The positioning of the grip size should be so that the second joint of the index finger is at a 90-degree angle of the handle (Figure 6). See here for more detail: https://www.youtube.com/watch?v=-jmWNKUek3o&t=58s.


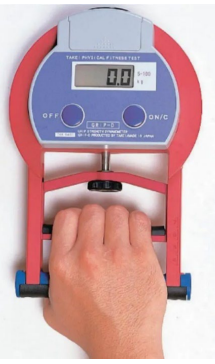


*Figure 6. Hand grip positioning*

- 1. Make a note of the handle position so this can be replicated for future tests.
  2. Start with the right hand and then repeat the measurement with the left hand (3 times per side).
  3. Read the measurement (in kg) from the dial and record the result.
  4. Disregard and repeat the test if the participant’s arm rises above the arm of the chair, or if their feet lift off the floor during the measurement.

1. Record three measurements for each hand, alternating sides and take an average.
2. If any scores are significantly lower (by >5kg) than the highest score on the same hand, exclude the lower scores from the average calculation. If two of the three efforts are >5kg lower than the other effort, record the highest score only.

Fatigue

Fatigue is measured using the Functional Assessment of Chronic Illness Therapy-Fatigue (FACIT-Fatigue), which has been validated for use with cancer patients a[1]. The FACIT-Fatigue subscale is a 13-item measure that assesses levels of patients self-reported fatigue and its impact upon daily activities and function.

Anxiety

Symptoms of anxiety are measured using the Generalised Anxiety Disorder-7 (GAD-7) questionnaire which consists of seven items [2].

Depression

Symptoms of depression are measured using the Patient Health Questionnaire-9 (PHQ-9), which assesses and monitors depression severity utilising a nine-item scale [3].

EVS

Exercise Vital Signs provides a self-reported proxy measure of physical activity in patients [4], recorded as the total minutes per week of physical activity based on the following questions:

1. On average, how many days per week do you engage in moderate to strenuous exercise (like a brisk walk)?

2. On average, how many minutes do you engage in exercise at this level?

Calculation = (1) x (2) = total minutes per week of physical activity = EVS.

Self-Efficacy for Exercise

Patient exercise self-efficacy is measured using the Self-Efficacy for Exercise scale (SEE) [5]. The SEE consists of nine situations (weather, boredom, pain, exercising alone, not pleasurable, too busy, feel tired, stress, depressed) that might affect participation in exercise. For each situation, the participant uses the scale from 0 (Not Confident) to 10 (Very Confident) to describe their current confidence that they could exercise 3 times a week for 20 minutes each time.

**Supplementary material 3 – Directed acyclic graphs**


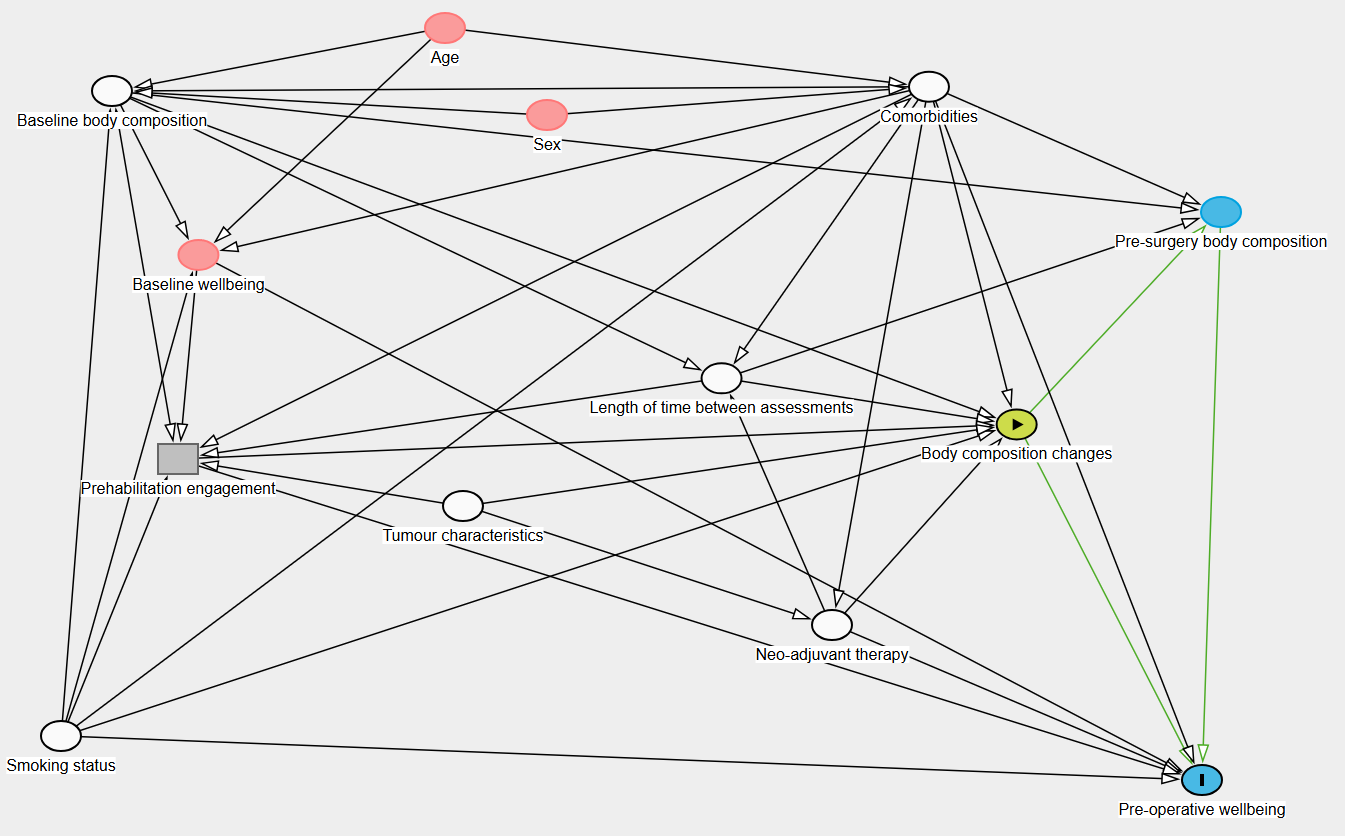


*Figure 9. Directed acyclic graph for investigating the effect of body composition changes on pre-operative wellbeing. Baseline and pre-operative wellbeing include nutritional status, psychological wellbeing, and physical fitness.*


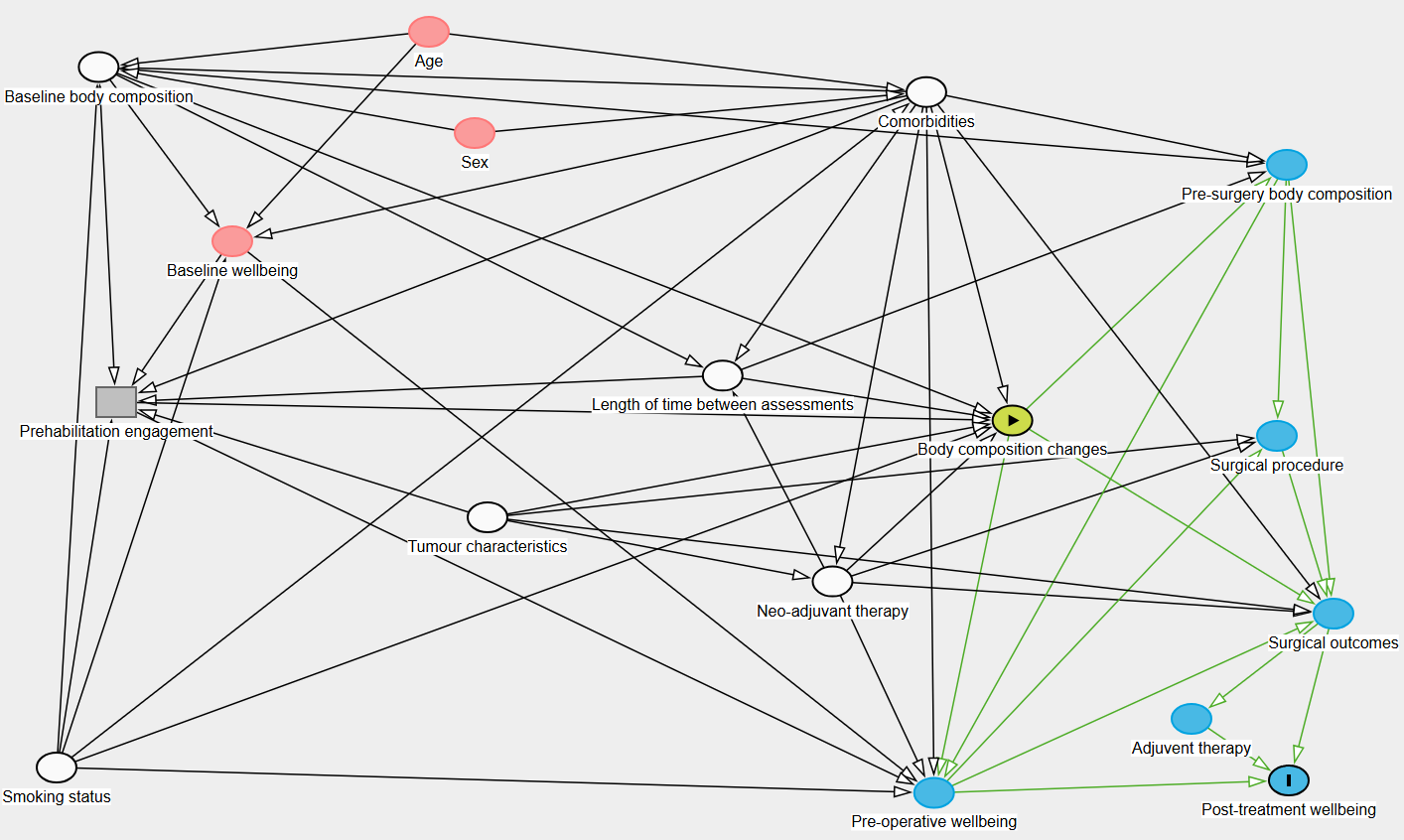


*Figure 10. Directed acyclic graph for investigating the effect of body composition changes on post-treatment wellbeing. Treatment includes any chemotherapy, radiotherapy and surgery. Baseline and pre-operative wellbeing include nutritional status, psychological wellbeing, and physical fitness.*


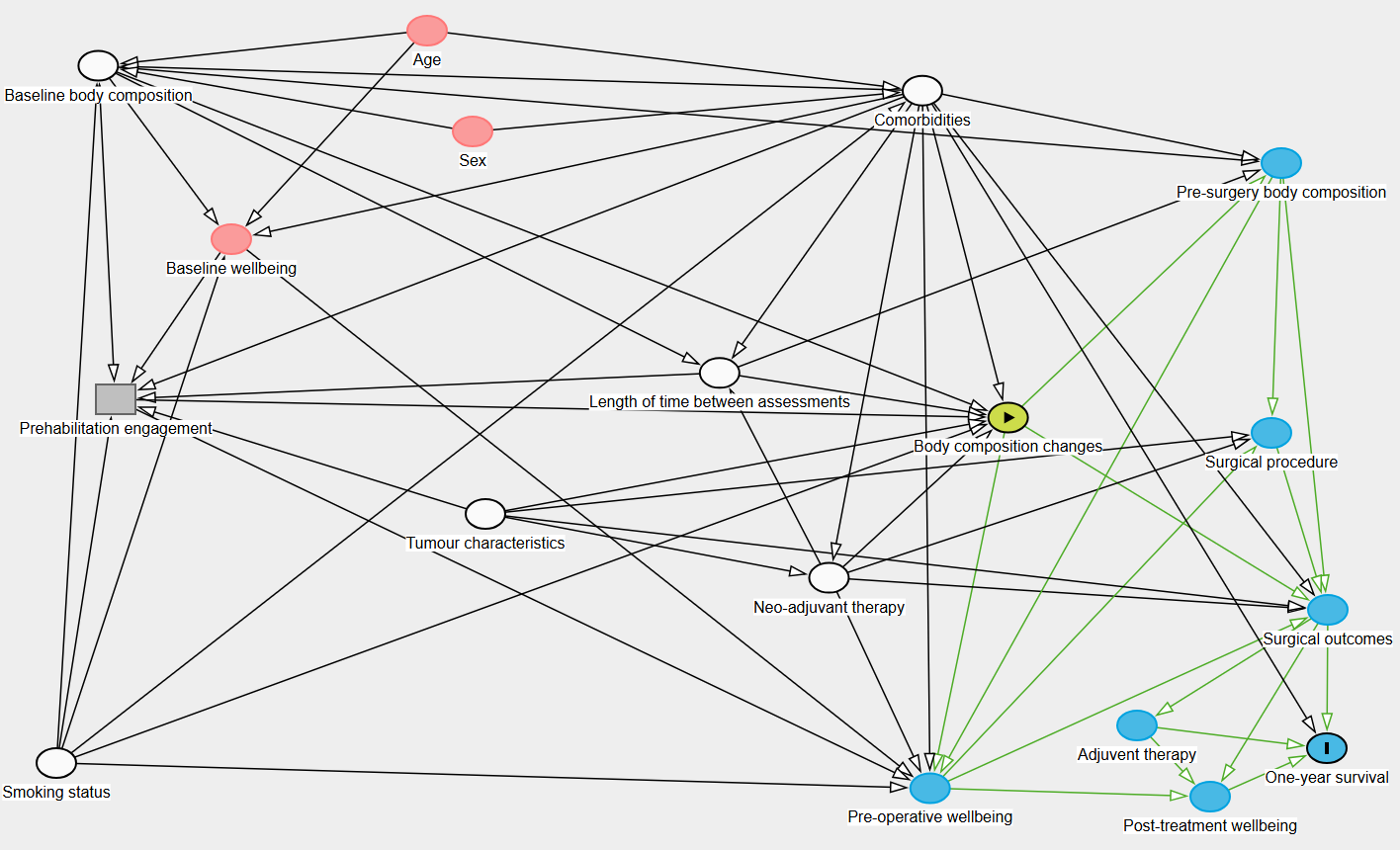


*Figure 11. Directed acyclic graph for investigating the effect of body composition changes on one-year survival. Baseline and pre-operative wellbeing include nutritional status, psychological wellbeing, and physical fitness.*

**References**

1. Acaster S, Dickerhoof R, DeBusk K, et al. Qualitative and quantitative validation of the FACIT‑fatigue scale in iron deficiency anemia. Health Qual Life Outcomes. 2015;13:60. doi:10.1186/s12955-015-0257-x.
2. Spitzer RL, Kroenke K, Williams JB, et al. A brief measure for assessing generalized anxiety disorder: the GAD‑7. Arch Intern Med. 2006;166(10):1092–7. doi:10.1001/archinte.166.10.1092.
3. Kroenke K, Spitzer RL, Williams JB, et al. The PHQ‑9: validity of a brief depression severity measure. J Gen Intern Med. 2001;16(9):606–13. doi:10.1046/j.1525-1497.2001.016009606.x.
4. Coleman K, Ngor E, Reynolds K, et al. Initial validation of an exercise “vital sign” in electronic medical records. Med Sci Sports Exerc. 2012;44(11):2071–6. doi:10.1249/MSS.0b013e3182630ec1.
5. Resnick B, Jenkins LS, et al. Testing the reliability and validity of the Self‑Efficacy for Exercise scale. Nurs Res. 2000;49(3):154–9. doi:10.1097/00006199-200005000-00007.
