# Supplementary material for: Associations between preoperative weight and body composition changes and surgical outcomes in patients with obesity and gastrointestinal cancer: a protocol for a prospective observational study in the active together cancer prehabilitation service
Source: BMJ Open. 2026 Jun 18;16(6):e120499. doi: 10.1136/bmjopen-2026-120499 (PMC13289135; doi:10.1136/bmjopen-2026-120499)
Supplement: Supplementary file 2 [file bmjopen-2026-120499.reviewer_comments.pdf]

PEER REVIEW HISTORY

BMJ Open publishes all reviews undertaken for accepted manuscripts. Reviewers are asked to complete a checklist review form and are provided with free text boxes to elaborate on their assessment. These free text comments are reproduced below.

ARTICLE DETAILS

Title (Provisional)

The associations between pre-operative weight and body composition changes and surgical outcomes in patients with obesity and gastrointestinal cancer: a prospective observational study protocol in the Active Together cancer prehabilitation service

Authors

Newbould, Scott; Myers, Anna; Rosenthal, Kerry; Harris, Keith; Thelwell, Michael; Copeland, Robert; Allott, Sade

---

This article was not externally reviewed at BMJ Open. Protocols that have been independently assessed prior to submission to BMJ Open are usually fast-tracked to publication on the grounds that further substantial changes will not be possible. This independent assessment will usually be external, independent review for both a substantial grant award from a non-commercial or government funder and ethics approval.
